# Supplementary material for: SRSF3 is oncogenic in breast but tumor-suppressive in liver by differential regulation of gene expression
Source: bioRxiv. 2025 Mar 17:2025.03.14.643315. Preprint. [Version 1] doi: 10.1101/2025.03.14.643315 (PMC12190909; doi:10.1101/2025.03.14.643315)
Supplement: Supplement 7 [file NIHPP2025.03.14.643315v1-supplement-7.pdf]

bioRxiv preprint doi: https://doi.org/10.1101/2025.03.14.643315; this version posted March 17, 2025. The copyright holder for this preprint (which was not certified by peer review) is the author/funder. This article is a US Government work. It is not subject to copyright under 17 USC 105 and is also made available for use under a CC0 license.

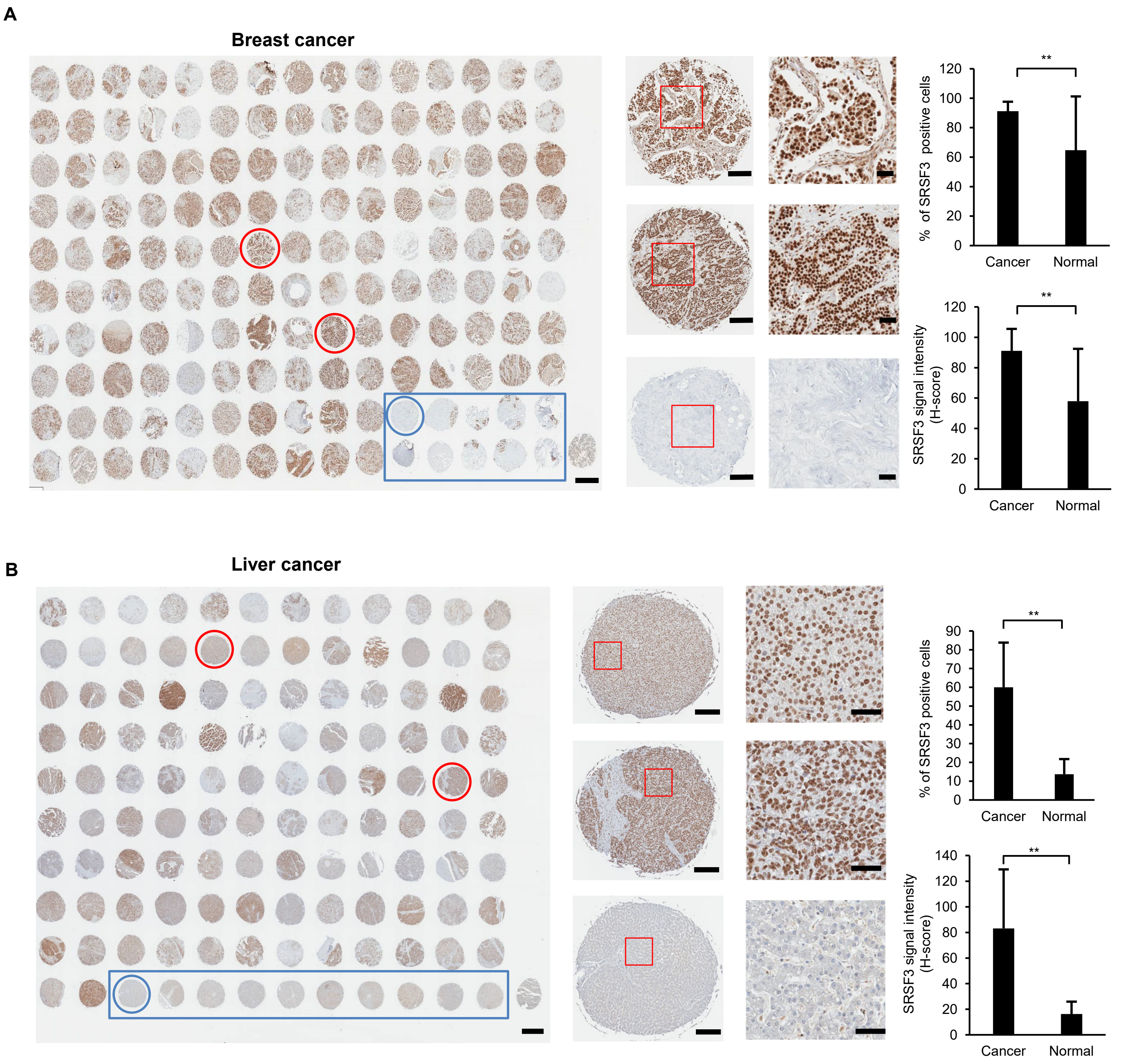

Fig. S1. SRSF3 overexpressed in both human breast cancer and liver cancer tissues. (A) SRSF3 immunohistology staining of 70 paired human breast cancer tissues and 5 paired normal tissues (tissue array information could be found in https://www.tissuearray.com/BR1504b). (B) SRSF3 immunohistology staining of 110 unpaired, single human liver cancer tissues and 10 normal tissues (tissue array information could be found in https://www.tissuearray.com/tissue-arrays/Liver/BC03119b). For both panel A and panel B, Scale bars from left to right, 1mm, 200μm, 50μm. Normal tissues were highlighted in blue rectangle. SRSF3 signal was quantified by HALO software (https://indicalab.com/halo/). Statistical comparisons were computed by unpaired, two-tailed Student's t test. ** p<0.01.

bioRxiv preprint doi: https://doi.org/10.1101/2025.03.14.643315; this version posted March 17, 2025. The copyright holder for this preprint (which was not certified by peer review) is the author/funder. This article is a US Government work. It is not subject to copyright under 17 USC 105 and is also made available for use under a CC0 license.

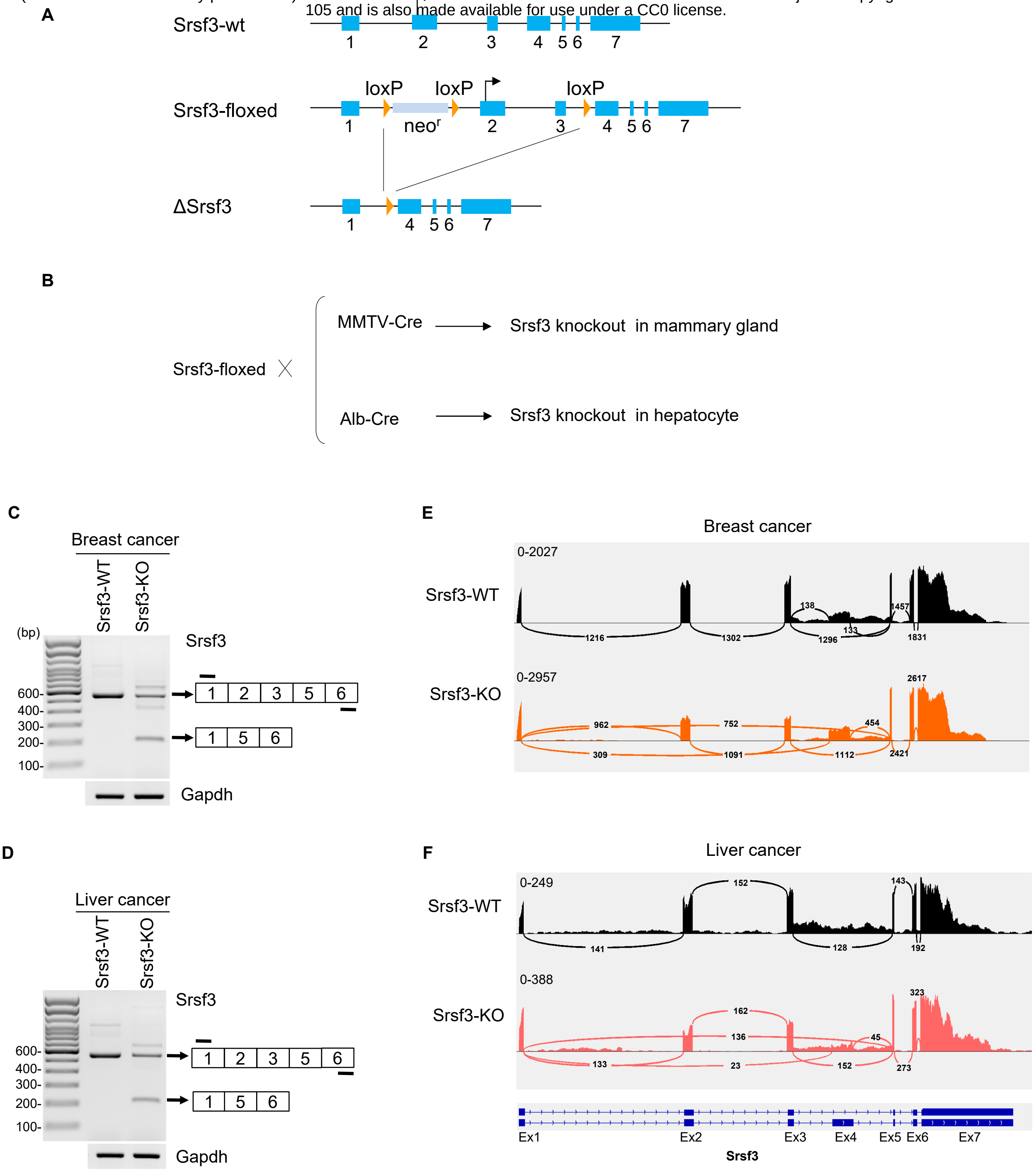

Fig. S2. Strategies implemented for tissue-specific conditional knock-out (KO) of mouse Srsf3 in breast and liver tissues. (A) Diagrams of Srsf3 gene structure and knockout strategy by Cre-loxP technology. Srsf3 gene consists of seven exons with the start codon AUG in the second exon. In the Srsf3-floxed allele (Srsf3-floxed) as described (Jumaa H., et al. Current Biol. 9: 899-902, 1999), loxP sequences are inserted in the first and third introns, leading to excise the Srsf3 exon 2 and 3 from genomic DNA when Cre recombinase is expressed in cells to induce Srsf3 knockout due to loss of start codon (ΔSrsf3). Floxed Neo^R, neomycin-resistant gene in the SRSF3 intron 1 under control by LoxP for cell selection. (B) Tissue-specific Srsf3 knockout is achieved by crossing Srsf3-floxed mice with Cre recombinase-expressing mice under a murine mammary tumor virus promoter (MMTV-Cre) or an albumin promoter (Alb-Cre), selectively active in mammary gland tissues or hepatocytes, respectively.(C and D) Sashimi plots showing the alternative splicing of Srsf3 gene in representative Srsf3 WT and KO breast cancer (C) and liver cancer tissues (D). The numbered arches represent splice junction reads detected from IGV. (E and F) RT-PCR validation for Srsf3 KO in breast cancer (E) and liver cancer tissues (F). Cre-LoxP recombination or KO of Srsf3 exons 2 + 3 mediated by tissue-specific Cre was confirmed by indicated primers from exon 1 and exon 6 (dash lines). GAPDH RNA served as a loading control.

bioRxiv preprint doi: https://doi.org/10.1101/2025.03.14.643315; this version posted March 17, 2025. The copyright holder for this preprint (which was not certified by peer review) is the author/funder. This article is a US Government work. It is not subject to copyright under 17 USC 105 and is also made available for use under a CC0 license.

A

Srsf3 KO (♀)

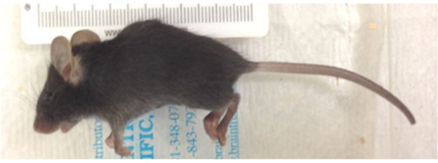

Srsf3 WT (♀)

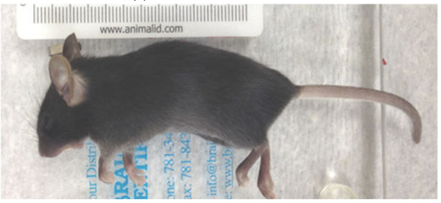

B

Early death incidence of the mice after DEN injection

genotype/gender	Incidence		Death days after birth (mean ± SE)
Srsf3 WT, DEN ♂ (n=76)	0/76	(0%)	N/D
Srsf3 hetKO, DEN ♂ (n=17)	0/17	(0%)	N/D
Srsf3 KO, DEN ♂ (n=38)	9/38*	(23.7%)	31.8 ± 1.7
Srsf3 WT, DEN ♀ (n=92)	0/92	(0%)	N/D
Srsf3 hetKO, DEN ♀ (n=19)	0/19	(0%)	N/D
Srsf3 KO, DEN ♀ (n=54)	13/54*	(24.1%)	37.5 ± 6.5

Fig. S3. Summary of early phenotypes of liver Srsf3-KO mice. (A) Pictures represent typical liver Srsf3-KO and WT female mice at 2 weeks after birth. (B) Early death incidence of Srsf3-WT, -hetKO, and -KO mice by days after one dose DEN injection on day 15 of age. *Some of the survived mice with DEN injection were sacrificed for serology and pathology examination in the middle term of the study.

bioRxiv preprint doi: https://doi.org/10.1101/2025.03.14.643315; this version posted March 17, 2025. The copyright holder for this preprint (which was not certified by peer review) is the author/funder. This article is a US Government work. It is not subject to copyright under 17 USC 105 and is also made available for use under a CC0 license.

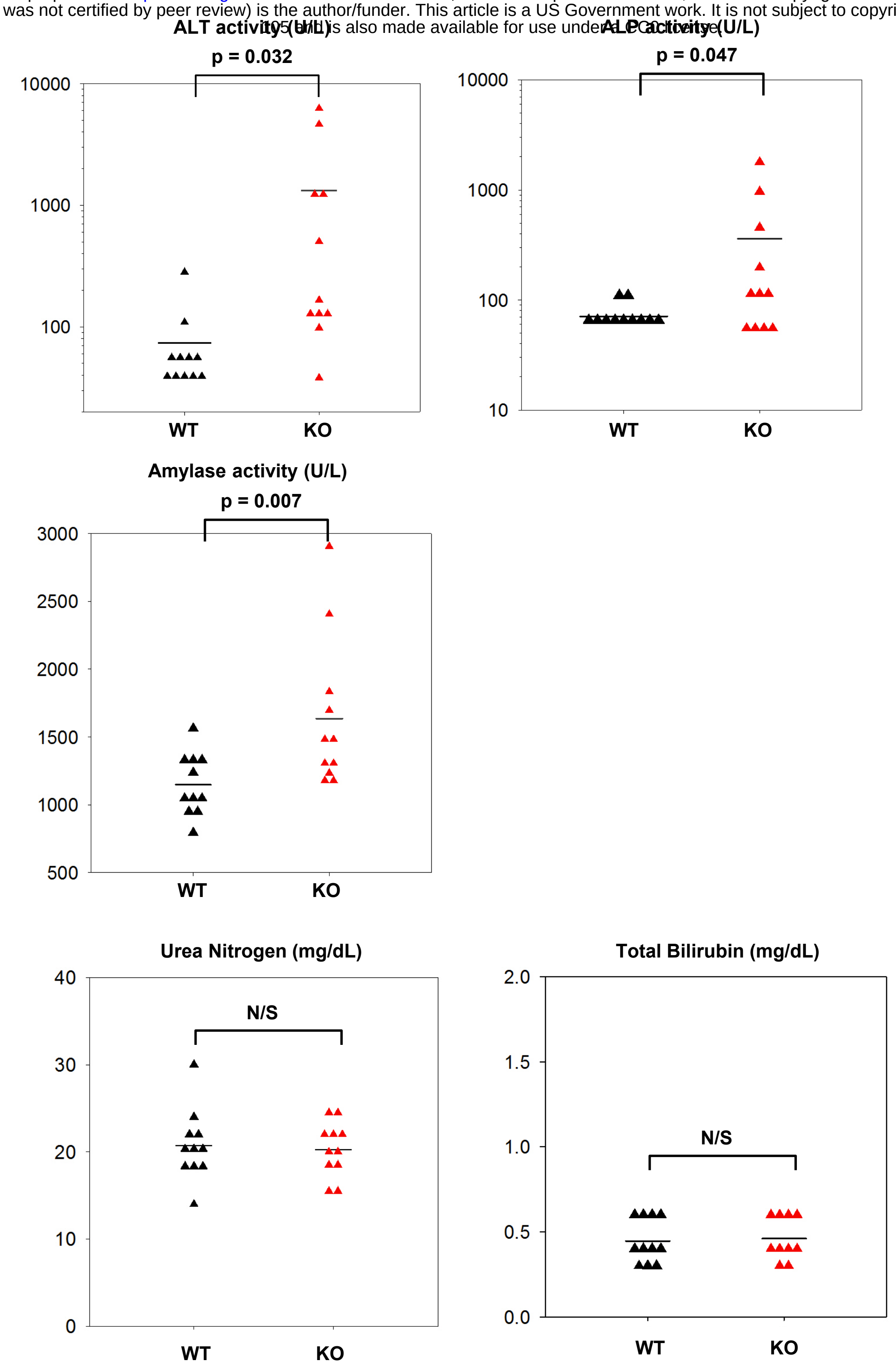

Fig. S4. Srsf3 KO affects mouse liver function changes in the 1-year-old animals. Blood samples were collected from DEN-injected Srsf3 WT and liver Srsf3 KO mice at a year of age and serum ALT, alkaline phosphatase, amylase activity, urea nitrogen and bilirubin were measured in a blood chemistry laboratory. A p-value was calculated by Student's t-test. N/S, not significant.

bioRxiv preprint doi: https://doi.org/10.1101/2025.03.14.643315; this version posted March 17, 2025. The copyright holder for this preprint (which was not certified by peer review) is the author/funder. This article is a US Government work. It is not subject to copyright under 17 USC 105 and is also made available for use under a CC0 license.

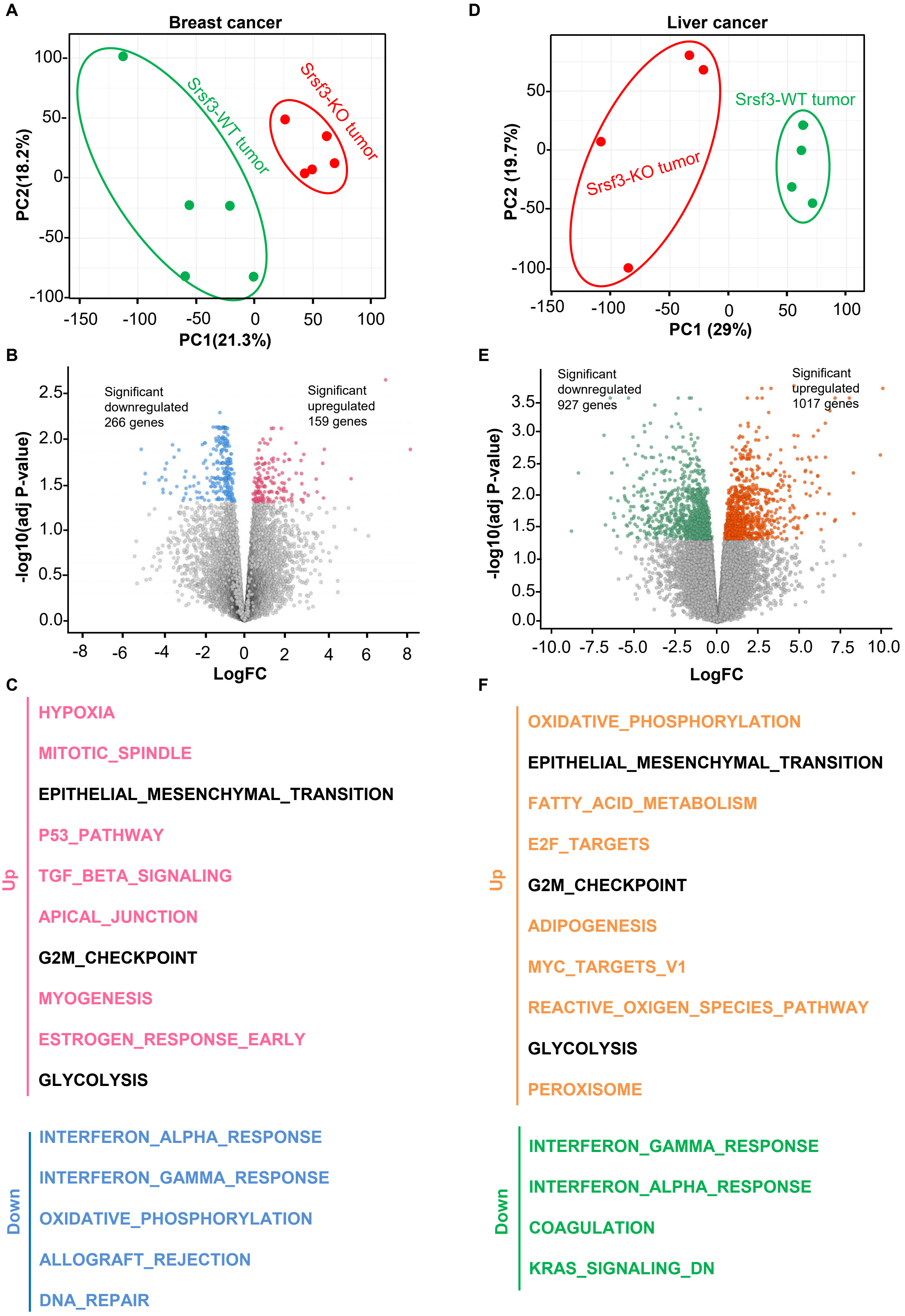

Fig. S5. RNA-seq analysis of Srsf3 associated genes in Srsf3-WT and Srsf3-KO breast and liver cancers. Principal component analysis (PCA) of Srsf3 WT (green) and Srsf3 KO (red) breast cancer (A) and liver cancer (D) by RNA-seq analysis. Five mammary gland cancer tissues and four liver tcancer tissues were submitted to RNA-seq analysis in each group. (B and E) Volcano plot showing the down-regulated and upregulated genes (adjusted $p \le 0.05$) in Srsf3 KO cancer compared to Srsf3 WT cancer. (C and F) Top activated and inhibited pathways by Gene Set Enrichment Analysis (GSEA) performed with the Hallmark gene sets.

bioRxiv preprint doi: https://doi.org/10.1101/2025.03.14.643315; this version posted March 17, 2025. The copyright holder for this preprint (which was not certified by peer review) is the author/funder. This article is a US Government work. It is not subject to copyright under 17 USC 105 and is also made available for use under a CC0 license.

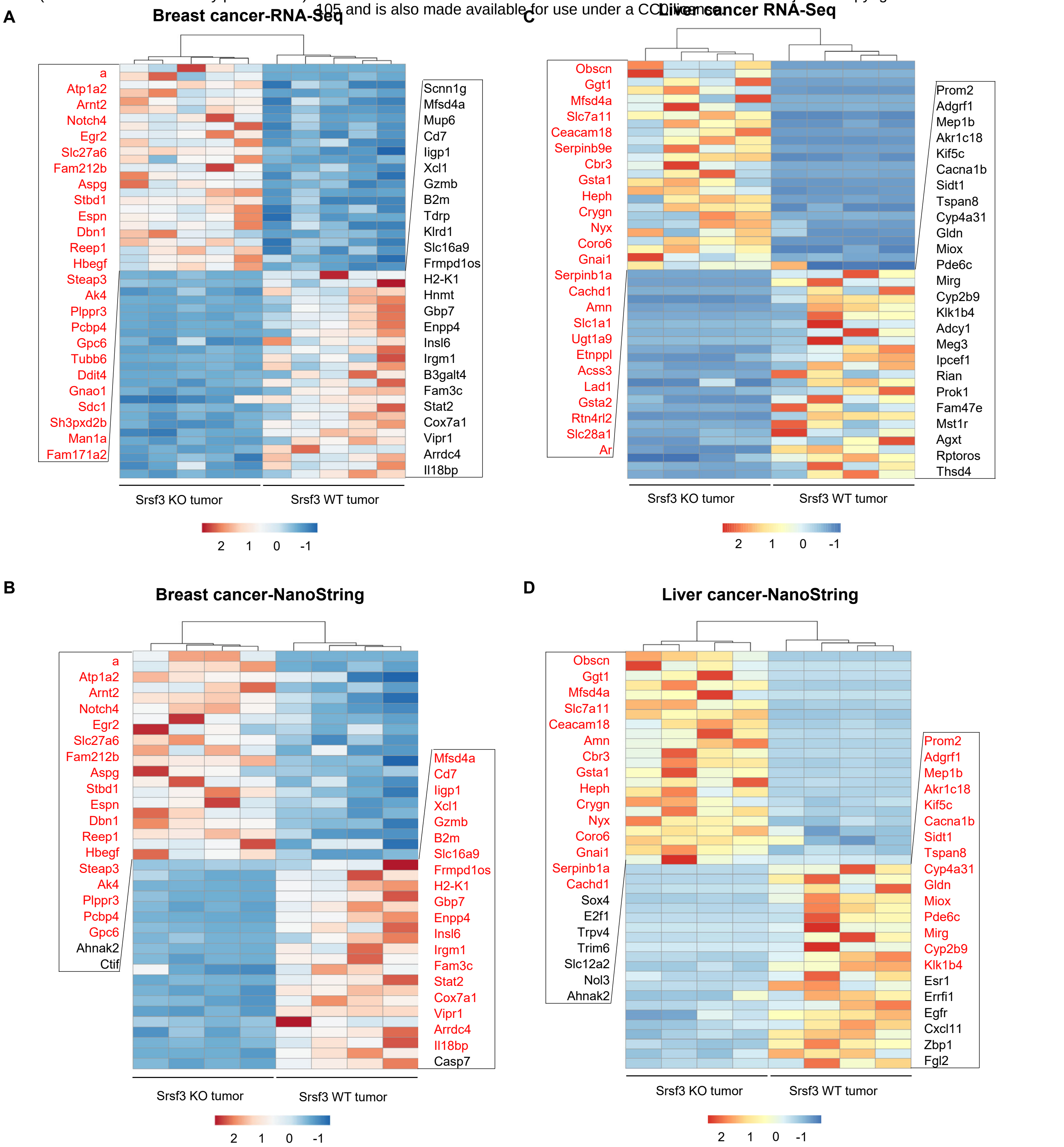

Fig. S6. Identification of the top Srsf3 regulated gens by RNA-seq and validation by NanoString analysis in breast cancer and liver cancer. (A and C) Hierarchical clustering and heatmap of top 25 genes significantly upregulated and downregulated by Srsf3 KO in breast cancer (A) (Adjusted $p \leq 0.05$, minRPKM >=2) and in liver cancer (C) (Adjusted $p \leq 0.05$, minRPKM >=5) by RNA-seq analysis. (**B** and **D**) Selective validation by NanoString technology of the RNA-seq identified top genes up- or down-regulated by Srsf3 KO in breast cancer (**B**) and in liver cancer (**D**). Genes highlighted in red in heatmap are the genes identified by RNA-seq and validated by NanoString.

bioRxiv preprint doi: https://doi.org/10.1101/2025.03.14.643315; this version posted March 17, 2025. The copyright holder for this preprint (which was not certified by peer review) is the author/funder. This article is a US Government work. It is not subject to copyright under 17 USC 105 and is also made available for use under a CC0 license.

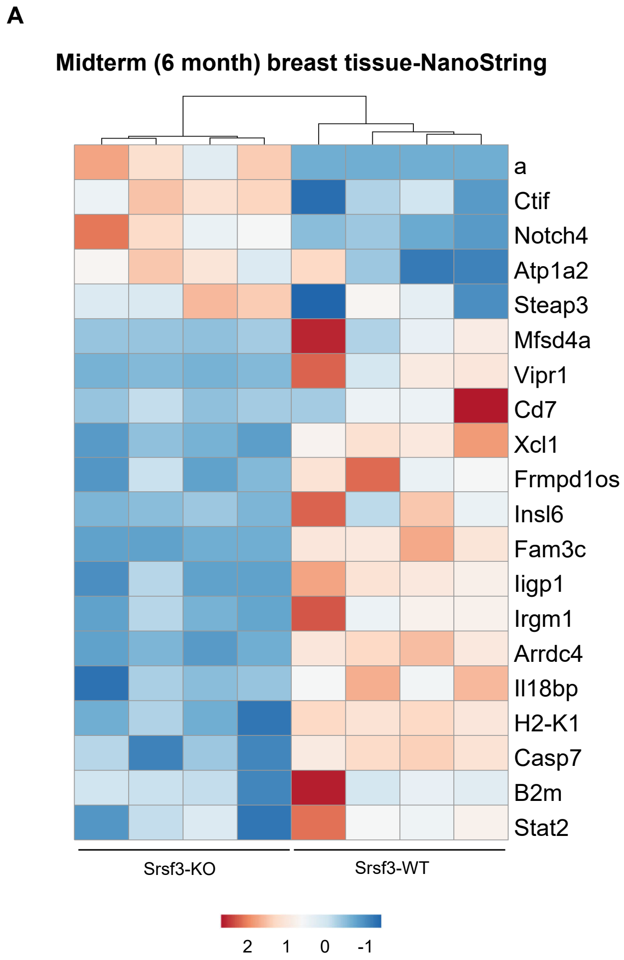

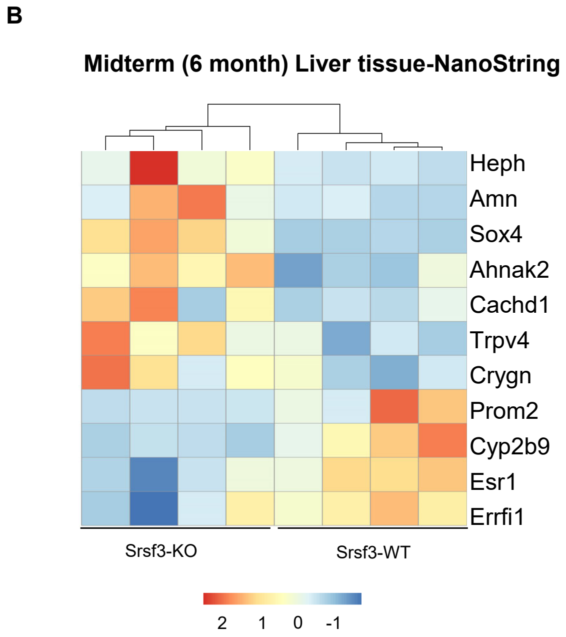

Fig. S7. Srsf3 KO in breast and liver tissues affects differential expression of a subset of genes. Total RNA isolated from the indicated mouse tissues with or without Srsf3 KO at six months of mouse age were used for NanoString RNA analysis. Mice donated liver tissues for this study were also treated by DEN injected at day 15 of age. Heatmap shows differential expression of Srsf3 target genes identified in breast (A) and liver (B) tissues at this stage of animals.

bioRxiv preprint doi: https://doi.org/10.1101/2025.03.14.643315; this version posted March 17, 2025. The copyright holder for this preprint (which was not certified by peer review) is the author/funder. This article is a US Government work. It is not subject to copyright under 17 USC 105 and is also made available for use under a CC0 license.

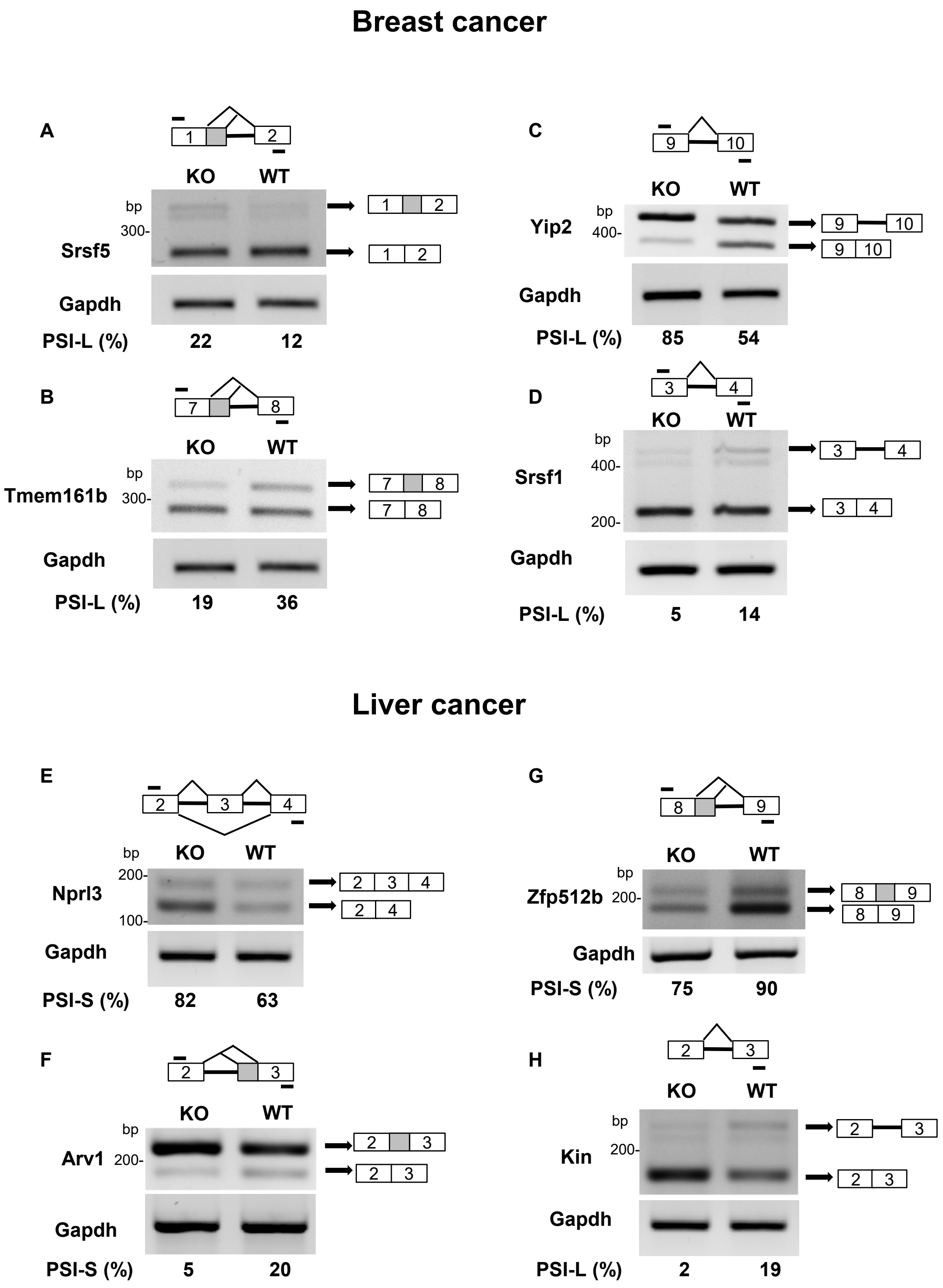

Fig. S8. Validation of significant splicing events in differential expression of the Srsf3 target genes from Srsf3-KO to Srsf3-WT breast cancer (A-D) and liver cancer (E-H). Total RNA from the Srsf3-WT or srsf3-KO cancer tissues were analyzed by RT-PCR to validate the altered splicing events identified by by rMATS (p<=0.05, FDR<=0.05). (A-D) Srsf3 KO in breast cancer tissues alters alternative 5′ ss usage in Srsf5 exon 1 (A) and in Tmem161b exon 7 (B) and affects intron retention of Yip2 intron 9 (C) and Srsf1 intron 3 (D). (E-H) Srsf3 KO in liver cancer tissues decreases Nprl3 exon 3 skipping (E) but increases alternative 3′ ss usage in the Arv1exon 3 (F), alternative 5′ ss usage in the Zfp512b exon 8 (G), and Kin intron 2 retention (H). The primers used in RT-PCR are shown as bars above (forward primers) and below (reverse primers) each pre-mRNA diagram. GAPDH served as a loading control. PSI, percent spliced-in of the alternative exon(s) or splice site (% inclusion = inclusion/sum of inclusion + exclusion).

bioRxiv preprint doi: https://doi.org/10.1101/2025.03.14.643315; this version posted March 17, 2025. The copyright holder for this preprint (which was not certified by peer review) is the author/funder. This article is a US Government work. It is not subject to copyright under 17 USC 105 and is also made available for use under a CC0 license.

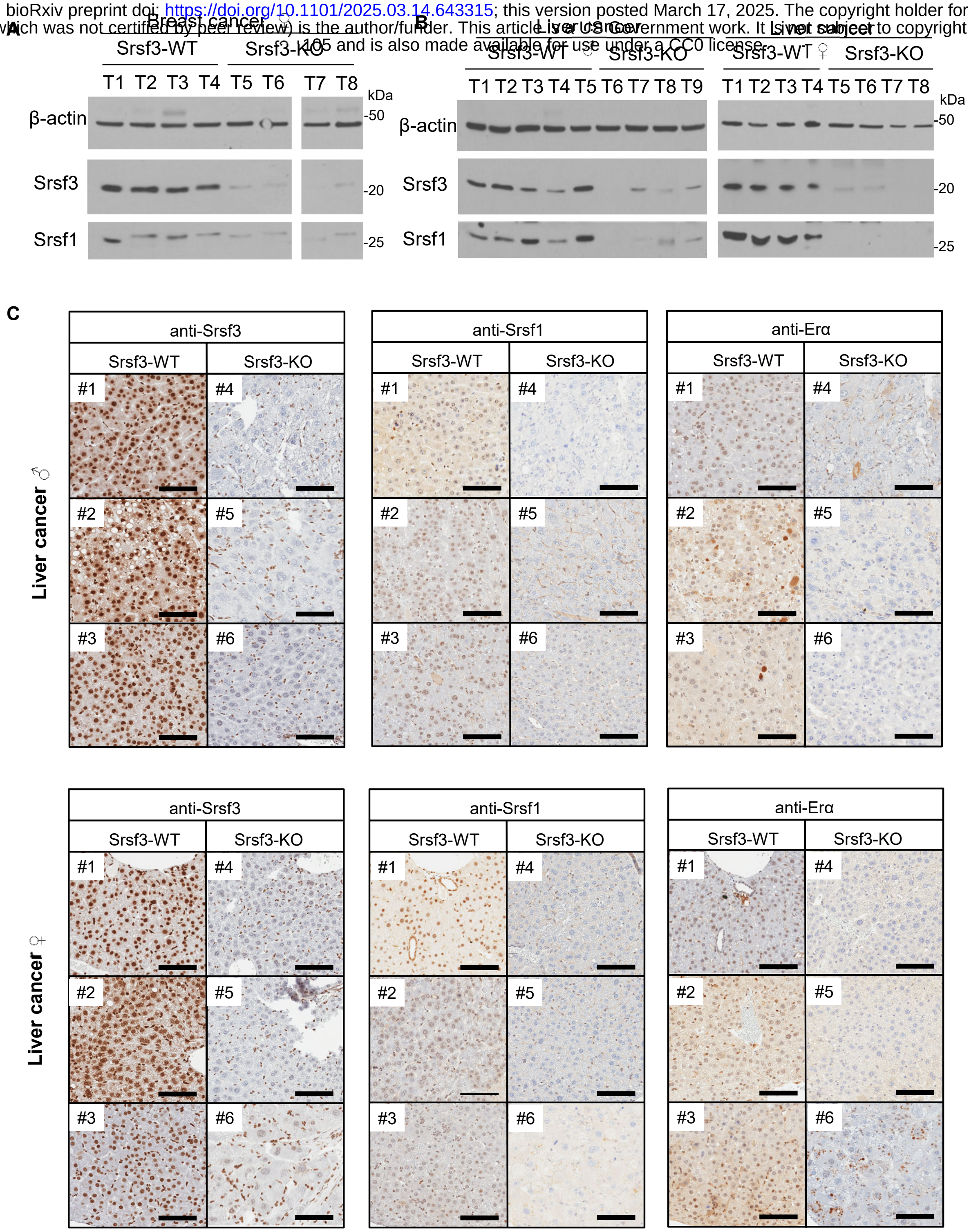

Fig. S9. Srsf3 KO decreased SRSF1 protein expression in both breast and liver cancers. (A and B) knockout of Srsf3 expression in breast cancer (A) and liver cancer (B) led to reduce the protein expression of SRSF1 by Western blot analysis. Minimal four cancer tissues in each group were used for the assay. β-actin served as a loading control. (C) Immunohistology staining of Srsf3, Srsf1and Erα protein in both male and female SRSF3-WT and -KO liver cancer tissues. Three pairs of representative samples in both genders were shown. Scale bar, 100 μm.

bioRxiv preprint doi: https://doi.org/10.1101/2025.03.14.643315; this version posted March 17, 2025. The copyright holder for this preprint (which was not certified by peer review) is the author/funder. This article is a US Government work. It is not subject to copyright under 17 USC 105 and is also made available for use under a CC0 license.

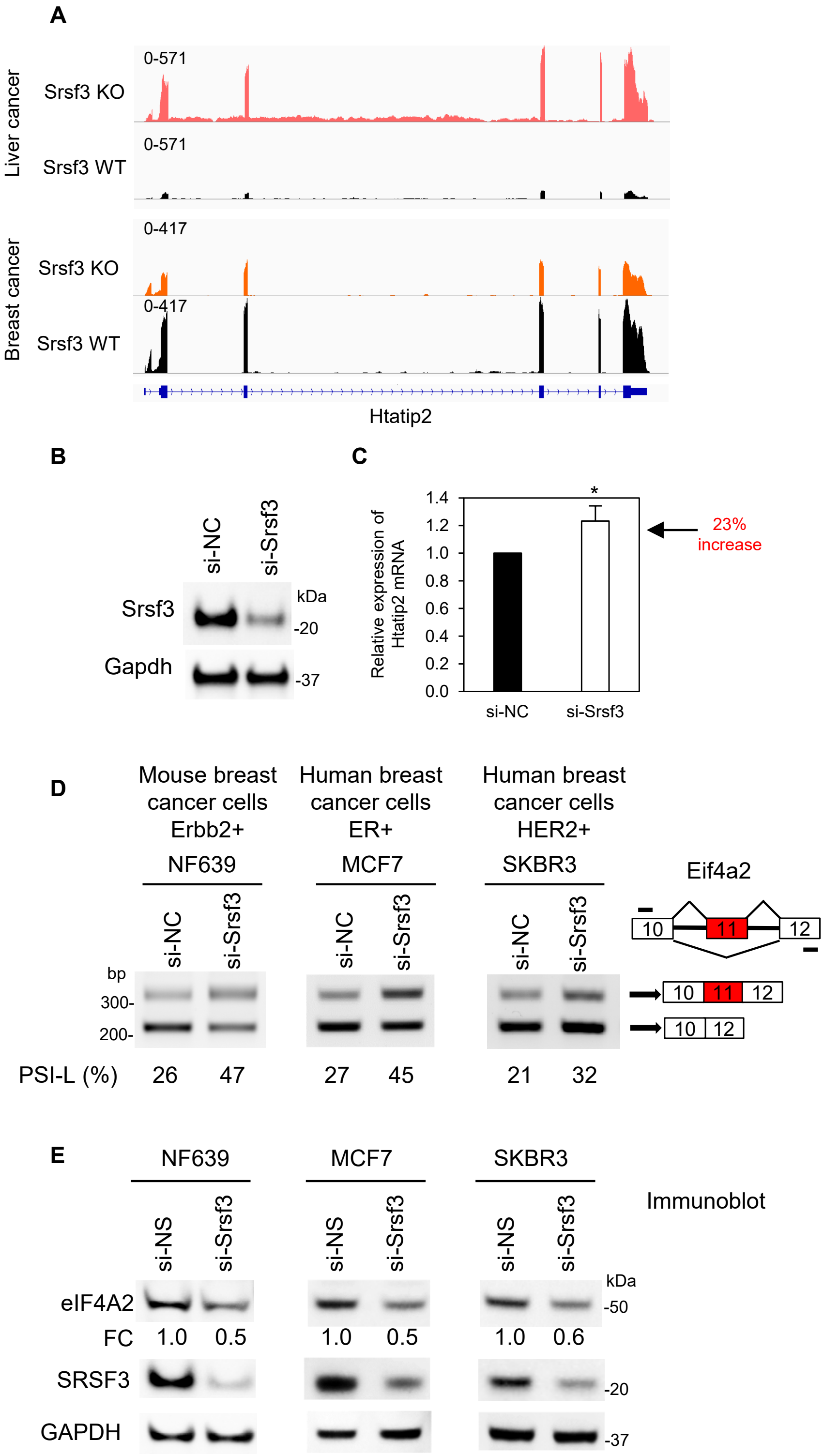

Fig. S10. Srsf3 knockout promotes Htatip2 expression in liver cancer but reduces Htatip2 expression in breast cancer visualized by IGV (A). Srsf3 KD in Hepa 1-6 cells, a murine hepatoma cell line, led to increased expression of Htatip2. Hepa1-6 cells were transfected with 40 nM of Srsf3-specific siRNA (si-Srsf3) or non-targeting siRNA (si-NC) and harvested at 48h upon transfection for analysis. Srsf3 KD efficiency was confirmed by Western blot, with Gapdh serving as a sample loading control (B). (C) Srsf3 KD in Hepa 1-6 cells led to increased expression of Htatip2 examined by TaqMan RT-qPCR in three independent experiments. *, p< 0.05 by Student's t-test.. (D-E) Knockdown (KD) of Srsf3/SRSF3 promotes exon 11 inclusion in Eif4a2 RNA splicing leading to reduction of eIF4A2 protein in mouse and human breast cancer cells. Total cell RNA was extracted from the cultured individual cell lines transfected with 40 nM of the indicated siRNAs and harvested at 48 h after transfection and used for RT-PCR assays with a forward primer from exon 10 and backward primer from exon 12 (D). Total cell extracts from corresponding cell lines prepared at the 48 h after siRNA transfection were immunoblotted for the protein expression of eIF4A2, SRSF3, and GAPDH with the corresponding antibodies (E).

bioRxiv preprint doi: https://doi.org/10.1101/2025.03.14.643315; this version posted March 17, 2025. The copyright holder for this preprint (which was not certified by peer review) is the author/funder. This article is a US Government work. It is not subject to copyright under 17 USC 105 and is also made available for use under a CC0 license.

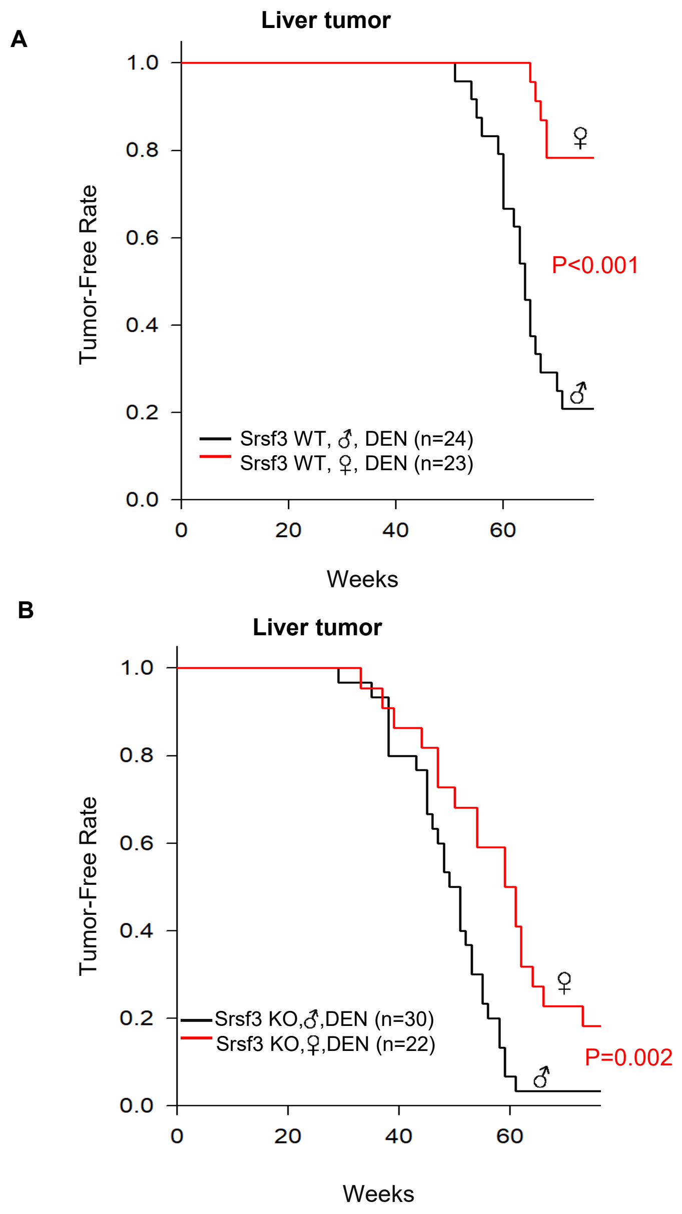

Fig. S11. Gender disparity in DEN- and DEN+Srsf3-KO induced liver tumor formation between male and female mice. Kaplan-Meyer plots were applied for the incidence of palpable tumors in Srsf3-WT (A) and Srsf3-KO mice (B) after DEN injection on day 15 of age. N= number of animals in each group. P-values were determined by log-rank test between indicated groups.

bioRxiv preprint doi: https://doi.org/10.1101/2025.03.14.643315; this version posted March 17, 2025. The copyright holder for this preprint (which was not certified by peer review) is the author/funder. This article is a US Government work. It is not subject to copyright under 17 USC 105 and is also made available for use under a CC0 license.

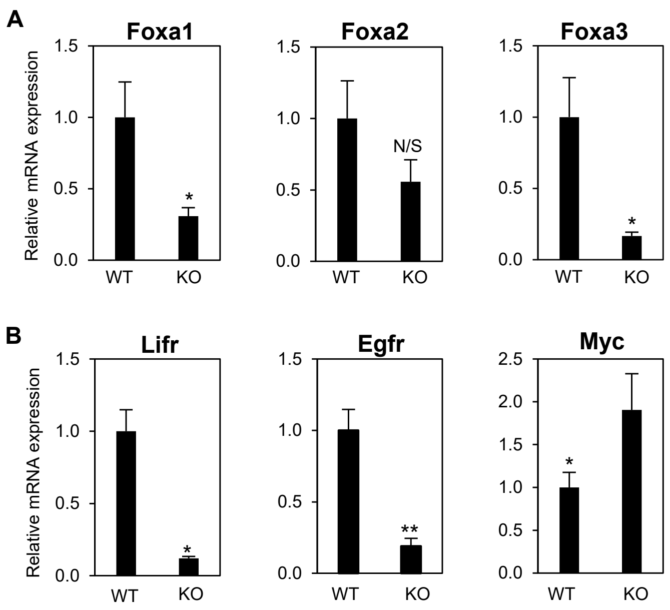

Fig. S12. RNA expression of Foxa1, Foxa 2, Foxa 3 (A) and Lifr, Egfr, Myc (B) in Srsf3-WT and Srsf3 KO male liver tumors by quantitative RT-qPCR. Five samples were in each group. N/S, not significant, *, $p < 0.05$; **, $p < 0.01$ by Student's t-test.
